# The Role of Maternal Thyroid Hormones in Avian Embryonic Development

**DOI:** 10.3389/fendo.2019.00066

**Published:** 2019-02-08

**Authors:** Veerle M. Darras

**Affiliations:** Laboratory of Comparative Endocrinology, Animal Physiology and Neurobiology Section, Biology Department, KU Leuven, Leuven, Belgium

**Keywords:** thyroid hormone, development, bird, deiodinase, TH transporter

## Abstract

During avian embryonic development, thyroid hormones (THs) coordinate the expression of a multitude of genes thereby ensuring that the correct sequence of cell proliferation, differentiation and maturation is followed in each tissue and organ. Although THs are needed from the start of development, the embryonic thyroid gland only matures around mid-incubation in precocial birds and around hatching in altricial species. Therefore, maternal THs deposited in the egg yolk play an essential role in embryonic development. They are taken up by the embryo throughout its development, from the first day till hatching, and expression of TH regulators such as distributor proteins, transporters, and deiodinases in the yolk sac membrane provide the tools for selective metabolism and transport starting from this level. TH receptors and regulators of local TH availability are expressed in avian embryos in a dynamic and tissue/cell-specific pattern from the first stages studied, as shown in detail in chicken. Maternal hyperthyroidism via TH supplementation as well as injection of THs into the egg yolk increase TH content in embryonic tissues while induction of maternal hypothyroidism by goitrogen treatment results in a decrease. Both increase and decrease of maternal TH availability were shown to alter gene expression in early chicken embryos. Knockdown of the specific TH transporter monocarboxylate transporter 8 at early stages in chicken cerebellum, optic tectum, or retina allowed to reduce local TH availability, interfering with gene expression and confirming that development of the central nervous system (CNS) is highly dependent on maternal THs. While some of the effects on cell proliferation, migration and differentiation seem to be transient, others result in persistent defects in CNS structure. In addition, a number of studies in both precocial and altricial birds showed that injection of THs into the yolk at the start of incubation influences a number of parameters in posthatch performance and fitness. In conclusion, the data presently available clearly indicate that maternal THs play an important role in avian embryonic development, but how exactly their influence on cellular and molecular processes in the embryo is linked to posthatch fitness needs to be further explored.

## Introduction

One of the major functions of thyroid hormones (THs), and probably also the most ancient one in vertebrate evolution, is coordinating embryonic and early postnatal development. By switching on and off the expression of a multitude of genes, THs ensure that the correct sequence of cell proliferation, differentiation, and maturation is followed in each developing organ/tissue. Many key developmental genes are only or mainly responsive to TH signaling during specific time windows in development. As a result, both untimely or too late expression of these genes may compromise the development and functioning of important organs in a persistent and irreversible way.

Although THs are needed from the start of development, early embryos lack a functional thyroid gland. This structure typically matures half way in embryonic development in precocial species such as chicken and Japanese quail, or only around the time of hatching in altricial species such as ring dove and red-winged blackbird (1–3). Mammalian embryos/fetuses can rely on a continuous supply of maternal THs via the placenta. External development in other vertebrates however precludes this possibility, so they rely on maternal THs deposited in the egg yolk. Early expression of TH distributor proteins, TH transporters, iodothyronine deiodinases, and TH receptors ensures that these hormones can be taken up from the yolk and transported into embryonic tissues where they can be activated and regulate gene transcription.

This review on the role of THs in avian embryonic development focuses on the role of maternal THs during early stages of development and explores the possible consequences for later posthatch life. Most of our current knowledge is derived from chickens where the embryonic thyroid gland is fully functional by mid-incubation (10-day-old embryo, E10) (1). Although the gland may already be able to secrete a small amount of THs a few days earlier, relevant contribution of embryonic THs to circulating levels is thought to start only around E8-E9 and to gradually increase thereafter up till hatching at E20. Fertilized chicken eggs are readily available and can easily be incubated in standard laboratory settings, facilitating experimental manipulation under controlled conditions throughout development. Moreover, the timing of the maturation of the thyroid axis is quite similar to that in the human fetus while maturation occurs much later in the classical rodent models. As a result, chicken is an excellent model system to study the role of THs in prenatal (human) development (4, 5). A limited number of data is also available for other economically relevant galliform species such as turkey and quail. Data on altricial species are scarce, and samples have been collected predominantly from songbird populations in the wild, where experimental manipulation is difficult and often not even allowed. These data are however important; since the thyroid gland of altricial species only matures late in incubation or even posthatch (6), maternal THs are the only source available throughout embryonic development.

## Maternal TH Content in Avian Eggs and Embryos

Since THs are lipophilic, only a small amount of maternal TH is found in the egg albumen while the vast majority is deposited in the yolk. Reported average levels for a number of avian species are summarized in Table 1. The concentration of 3,5,3′,5′-tetraiodothyronine or thyroxin (T_4_) in yolk always exceeds that of 3,5,3′-triiodothyronine (T_3_), as found in almost all other oviparous vertebrates investigated so far. Interestingly, reported levels not only vary between species but also within species. In the case of chicken this is not surprising since the different commercial strains have been separated by long term selection. Reported average levels vary 10-fold for T_4_ and 3-fold for T_3_, showing differences between broilers and layers (10) but also between different layer strains (7, 9, 10). Variation however also occurs between individuals of a given species. In a recent study on songbirds, T_4_ levels were found to vary 3- to 4-fold, and T_3_ levels 2- to 3-fold, between individuals of the same species (11). Moreover, factors such as laying sequence, temperature and food availability induce changes in maternal TH deposition, with potential consequences for the offspring (14).

**Table 1:** Maternal TH content deposited in egg yolk of different avian species.

**Species**	**T_**4**_ in ng/g^a^**	**T_**3**_ in ng/g^a^**	**T_4_/T_3_^b^**	**References**
**PRECOCIAL BIRDS**				
**Chicken (*****Gallus gallus*****)**				
- Shaver strain (layer)	3.80 ± 0.81	1.50 ± 0.39	2.13	(7)
- Ross strain (broiler)	14.60 ± 2.24	1.23 ± 0.20	9.93	(8)
- Hy-line strain (layer)	30.4	1.0	25.4	(9)
- White leghorn strain (layer)		1.05 ± 0.36		(10)
- Cornish rocks strain (broiler)		0.46 ± 0.38		(10)
Japanese quail (*Coturnix japonica*)	9.74 ± 2.20	3.44 ± 0.88	2.38	(11)
	6	2.5	2.01	(12)
**ALTRICIAL BIRDS**				
Rock pigeon (*Columbia livia*)	3.06 ± 0.99	1.10 ± 0.21	2.33	(13)
Great tit (*Parus major*)	1.15 ± 0.42	0.14 ± 0.07	6.73	(11)
Collared flycatcher (*Ficedula albicollis*)	7.21 ± 0.99	1.97 ± 0.48	3.06	(11)
Pied flycatcher (*Ficedula hypoleuca*)	5.72 ± 1.42	1.86 ± 0.56	2.57	(11)

a *Average concentrations ± SD are given when information is available*.

b *Ratio calculated on a molar basis*.

The amount of THs deposited in the egg in general varies in parallel with hormone levels in the maternal circulation: induction of maternal hypothyroidism decreases while maternal hyperthyroidism increases yolk TH levels (8, 12). However, females seem to be able to regulate TH deposition to some extent (13, 15), although the mechanisms by which this occurs are not yet understood. It was found in euthyroid Japanese quail that T_4_ content in individual eggs of a given hen was relatively constant despite fluctuations in plasma T_4_ (15). When quail hens were made hyperthyroid by two different doses of T_4_, both T_4_ and T_3_ content in eggs was significantly increased with both doses although plasma T_3_ was only increased following treatment with the higher dose (12). When hens were supplemented with T_3_, plasma T_3_ was increased and plasma T_4_ decreased but TH content in eggs remained stable (12). On the other hand, when chicken hens were made hypothyroid by goitrogen treatment, plasma T_4_ and T_3_ initially decreased but plasma T_3_ returned to normal by 14 weeks of treatment. Nevertheless, both T_4_ and T_3_ levels in egg yolk remained severely decreased (8).

To perform any function, maternal THs of course must be taken up by the developing embryo. It was found in chicken that significant amounts of TH were already released by area opaca cells before the start of gastrulation and that active TH (T_3_) was enriched in the primitive streak and Hensen's node during gastrulation (16). A longitudinal study in chicken by Iwasawa and coworkers from E4 till hatching showed that total yolk weight and total yolk T_4_ and T_3_ content decreased in an almost linear and parallel way throughout embryonic development (9), suggesting that the yolk transfers THs together with other components to the embryo in a continuous and non-selective way. However, TH measurements in head and trunk in embryos from hypothyroid chicken hens showed that the situation is more complex. Despite severely reduced T_4_ and T_3_ content in yolk, T_4_ levels in head and trunk of E6 embryos were normal and T_3_ levels were only reduced in head (8). This indicates that regulatory mechanisms exist, certainly within the embryonic tissues themselves (see next section), but probably also already at the level of the yolk sac membrane. Both transthyretin (TTR) and albumin (ALB) are expressed there and may contribute to TH transfer to the embryonic circulation (9). Next to these TH distributor proteins, yolk sac membrane also expresses the TH transporters monocarboxylate transporter 8 and 10 (MCT8, MCT10) and organic anion transporting protein 1C1 (OATP1C1) as well as all three types of deiodinases (DIO1, DIO2, DIO3) (9). Expression profiles are gene-specific and dynamic throughout embryonic development. The yolk sac membrane may therefore act as a selective barrier, similar to the mammalian placenta where DIOs and TH transporters are also dynamically expressed to regulate TH transfer to the fetus (17, 18).

Direct measurement of T_4_ and T_3_ in chicken embryonic tissues during the first days of development is difficult due to the limited sensitivity of the currently available methods, but it was shown indirectly by a reporter system that active TH was already present in blastoderm and subsequently also in the primitive streak (16). From E4 onwards, extraction of either whole embryos or brain tissue, followed by sensitive radio-immunoassay, allowed to directly determine the amount of both T_4_ and T_3_ (7, 19). Uptake of TH from egg yolk into embryonic tissues was additionally confirmed by an increase of T_3_ in E3 whole embryo extracts following injection of T_3_ into the yolk at E1 (Van Herck & Darras, unpublished results). Similarly, a combined T_4_+T_3_ injection into the yolk of embryos from different stages (E3-E11) increased levels of both hormones in extracts from brain collected 24 h later (E4-E12) (19). Maternal THs are therefore present in the early chicken embryo and can be active if the mediators of TH action are also available (see next section). Maternal THs were also shown to be present in plasma of altricial species such as ring dove and European starling where sampling was done at later stages of embryonic development but still prior to the presumed timing of thyroid gland maturation (20, 21).

## Regulators of TH Action at Early Stages

The majority of TH signaling occurs via binding of T_3_ to nuclear thyroid hormone receptors (THRs), so their presence is essential for TH action. Important in the context of development however is the fact that THRs can also function in an unliganded state, in most cases switching from active gene repression to stimulation of gene transcription upon ligand binding (22). Chickens express three isoforms of THRs: THRα, THRβ0, and THRβ2 (23). Messenger RNA for *THRA* is already present at the gastrula stage and *in situ* hybridization (ISH) showed that during neurulation, *THRA* is strongly expressed in the neural tube (16). At later stages and throughout development, this receptor is expressed in a wide array of embryonic tissues (24). Expression of *THRB* starts somewhat later in development and is mainly restricted to brain, eye, lung, kidney and yolk sac (24). Quantitative reverse transcription polymerase chain reaction (qRT-PCR) analysis of different brain regions at E4 and E8 revealed a dynamic and region-specific expression pattern for all three *THR* isoforms (19). It was also shown that the *THRA* mRNA present during neurulation was indeed translated to functional receptor protein since injection of high doses of T_3_ interfered with normal neural tube morphogenesis (16). Therefore, we can assume that TH signaling in the chicken embryo starts from the first day of development.

As THRα seems to be ubiquitously expressed from early stages (16, 25), the presence of additional regulators is essential to control local T_3_ availability and thereby coordinate the switch between unliganded and liganded THR function in a time- and tissue-dependent manner. Iodothyronine deiodinases (DIOs) are intracellular enzymes capable of activating and inactivating THs. DIO1 can stimulate both pathways but with relatively low affinity and is presently thought to be less important in euthyroid conditions (26). The main enzyme for local TH activation (T_4_ to T_3_ conversion) is DIO2 while DIO3 is the main enzyme for local TH inactivation (conversion of T_4_ to reverse T_3_ and T_3_ to 3,3′-diiodothyronine or 3,3′-T_2_) (26, 27). The earliest ISH data are from chicken brain at E3 showing *DIO2* expression in the hypothalamic region and *DIO3* expression in the mesencephalon and the eye (28, 29). Further ISH studies up to E10 showed increasing expression of *DIO2* throughout the brain and adenohypophysis and in the eye and inner ear. *DIO2* was abundantly expressed in endothelial cells of blood vessels throughout the brain, suggesting that in chicken T_4_ to T_3_ conversion may already occur at least partially just prior to entry into the brain at the level of the blood-brain-barrier (28). *DIO3* in contrast was highly expressed in the choroid plexus (28, 30), suggesting that the amount of active TH that can reach the cerebrospinal fluid is strictly controlled. Up to E10, *DIO3* mRNA was also found in some sensory brain centers and in the eye, while it was absent from the inner ear (28).

Using the more sensitive qRT-PCR as an alternative approach, mRNA of all three *DIOs* was detected in extracts from whole chicken embryos at E1 (31) and in extracts from brain sampled from E4-E12 (19). Next to the different expression dynamics in telencephalon, diencephalon, mesencephalon and rhombencephalon, the latter study also showed that *DIO2* and *DIO3* mRNA were translated into active enzymes while this was apparently not the case for *DIO1* (19). *DIO2* mRNA and activity were also reported in chicken brain by another research group in a study starting at E7 (32). Information on expression patterns of DIOs in peripheral tissues of chicken embryos during the first half of incubation is surprisingly scarce A relatively old report demonstrated the presence of both outer and inner ring deiodinating activity in liver at E8 (33) and one more recent ISH study showed *DIO1* mRNA at E4 in developing limb bud muscles and *DIO3* mRNA at E3/E4 in mesonephros/somites (29).

Before intracellular (in)activation by DIOs can occur, THs need transporters to facilitate their entry (and exit) through the plasma membrane. Chickens express four different TH transporters: MCT8, MCT10, OATP1C1, and L-type amino acid transporter 1 (LAT1), while LAT2 seems to be absent (34). The characteristics of these four chicken TH transporters are quite similar to those found in humans and zebrafish. MCT8 transports both T_4_ and T_3_ with high affinity, while MCT10 and OATP1C1 show a preference for T_3_ and T_4_ transport respectively. LAT1 has a lower affinity but is able to transport T_4_ and T_3_ next to its preferred iodothyronine, 3,3′-T_2_ (34). Messenger RNA for *MCT8* was already detectable by ISH at E1 where expression initially occurred in all three germ layers and subsequently shifted to foregut, head region and neural tube. Expression in the endoderm disappeared at E2 (35). In E3-E4 brain, *OATP1C1* signal was mainly found in the hypothalamic region while *MCT8* staining was more widely spread. Expression of *MCT8* continued to expand throughout the brain at E6-E10 but *OATP1C1* mRNA became more restricted with a strong signal in the adenohypophysis and median eminence (28, 34). *MCT8* but not *OATP1C1* mRNA was also found in the developing eye and inner ear (28).

In relation to the brain barriers, both *MCT8* and *OATP1C1* mRNA were found in the choroid plexus but absent from the endothelial cells at the blood-brain-barrier (28, 34). The only transporter detected by ISH in these cells was *LAT1*. Immunostaining revealed the presence of LAT1 protein both in the luminal and abluminal membrane of the endothelial cells, suggesting that despite its lower affinity, this transporter is an important regulator of T_4_ and T_3_ entry into the developing chicken brain (5, 34). *LAT1* was also expressed in blood vessels in spinal cord and in some cell types of eye while *MCT10* mRNA was found in eye and to some extent also in the choroid plexus (34). According to the same study, neither of the four transporters was expressed in sufficient amounts in heart, lung, intestine, liver, kidney, and gonads to be detectable by ISH at E10. At that stage positive staining in peripheral tissues was only found in pancreas for *LAT1* and *MCT10* and in feather buds for *LAT1* and *MCT8* (34). qRT-PCR data are only available for *OATP1C1* and *MCT8* expression in brain, showing a strong decrease in *OATP1C1* mRNA in telencephalon and diencephalon from E4 toward E10 while its expression in mesencephalon and rhombencephalon was stable and low. In contrast, *MCT8* expression gradually increased in all brain regions within the same time frame (19).

To have an efficient regulator function, one typically expects the above mentioned players to react to changes in maternal TH availability in the yolk in some sort of feedback system. Injection of THs (T_4_+T_3_) into the yolk at E3 indeed resulted in clear changes in brain *OATP1C1, MCT8, DIO2, THRA*, and *THRB* expression 24 h later. Surprisingly, this was no longer the case following injection at E7 (19). Although the results at E4 indicate responsiveness of these regulators at early stages, not all changes fit with what one would expect from a typical negative feedback response. Several of them are in line with the normal ontogenetic pattern (19), so they could at least partially be the result of a TH-induced acceleration of development. This shows that although TH regulators respond to TH status at early stages of development, the negative feedback system is still immature, making early embryos extra vulnerable for inadequate maternal TH supply.

In contrast to the substantial amount of data available for chicken (brain), data on expression of TH regulators in early embryos of other precocial avian species are lacking. With regard to altricial species, some information is available for ring dove just before and around the stages where the thyroid gland becomes active (6). *In vitro* testing of hepatic T_4_ to T_3_ conversion in the perinatal and early posthatch period showed that considerable outer ring deiodinating activity (probably DIO1) was present in embryos shortly before hatching while inner ring deiodinating activity (probably DIO3) may be less important in nestling doves as compared to embryonic quail (6, 20). However, neither DIOs nor TH transporters had been cloned and fully characterized at the time of these studies. Therefore, the only conclusion that can be drawn from them is that altricial embryos also take up maternal THs from the yolk and are capable of TH activation and inactivation.

## Effects of Maternal Hyper- and Hypothyroidism on Embryonic Development

Different methods have been used to alter maternal TH supply to chicken embryos. One way to increase TH availability in the yolk is to supplement laying hens with T_4_ and/or T_3_. Only a few studies followed this approach. Two papers report on a study with T_4_ supplementation in broiler breeder hens. They showed an increase in plasma T_4_ but not T_3_ of embryos at E18 and at internal pipping (36, 37), but embryos were not studied in more detail. Some information on effects in early development is available from a study where Japanese quail hens were dosed twice daily with 3x the daily thyroid gland secretion rate of T_4_, resulting in an accelerated growth and differentiation of embryonic pelvic cartilage, shown by its increased weight and alkaline phosphatase activity at day 9 of the 16 days incubation period (12).

A more easy alternative to maternal supplementation is to inject T_4_ and/or T_3_ directly into the yolk of fertilized eggs, allowing to control precisely the administered dose and time of injection. Injecting 1 μg T_4_ + 0.5 μg T_3_ into the yolk of chicken eggs at different stages (between E3-E11) always resulted in increased levels of both hormones in embryonic tissues 24 h later [(19) and Van Herck and Darras, unpublished results]. As mentioned before, this induced changes in the expression of TH regulator genes in embryonic brain at early stages (19). We took the same approach some years ago, injecting 0.5 μg T_3_ at E3 or E7, to analyze the brain transcriptome 48 h later using the chicken 44K microarray from Agilent (38). Statistical analysis revealed 187 differentially expressed genes at E5 and 420 differentially expressed genes at E9 (Van Herck and Darras, unpublished results). Next to gene ontology analysis, gene network analysis was performed with Ingenuity Pathway Analysis using the Genbank identities of the corresponding human proteins. The top gene interaction network identified at E5 (25 genes) was “Developmental disorders, Endocrine system disorders, Neurological disease” and at E9 (26 genes) “Cellular development, Hematological system development and function, Hematopoiesis.” Although these results are preliminary and a larger study is needed for full analysis (nowadays rather by RNA sequencing), they clearly prove that increasing “maternal” TH deposit influences biological processes during early brain development.

Injection of THs at the start of incubation has also been done in turkey, another precocial species, resulting in decreased hatchability (39). This differs from two recent studies in altricial species, showing enhanced embryonic development and hatching success in rock pigeon but not in great tit (40, 41). Unfortunately, none of these studies provided data on earlier embryonic stages.

In relation to TH deficiency, injection of goitrogens such as 2-mercapto-1-methylimidazole (MMI), 6-propyl-2-thiouracil (PTU) or ammonium perchlorate (AP) into the egg can block the embryonic thyroid gland and decrease TH availability, but only at stages where the thyroid gland starts contributing to circulating TH levels. Blocking conversion of maternal T_4_ into T_3_ at earlier stages by injecting PTU or iopanoic acid is also not an option since the high concentrations needed to efficiently block DIO1/DIO2 activity *in vivo* are toxic for the embryo (own observations). Decreasing maternal TH availability throughout development can only be achieved by rendering laying hens hypothyroid, which is typically done by addition of goitrogens to their food or drinking water. Finding the right dose can be a challenge since too mild maternal hypothyroidism does not sufficiently decrease yolk TH content while too severe hypothyroidism results in a reduction or even complete stop of egg laying as found in both quail and chicken [(12, 42) and own unpublished results].

Addition of 0.03% of MMI in drinking water of broiler breeder hens reduced the number of eggs with 70% by week 8. Yolk T_4_ and T_3_ content of eggs collected between week 10 and 16 were reduced with 70 and 50% respectively. Overall egg quality (egg weight, crude energy content, crude protein content and crude lipid content) was unaffected (8), precluding non-specific effects on embryonic development caused by nutrient deficiency. Morphological scoring of the embryos at E4, E6, and E8 according to the Hamburger and Hamilton stages (43) suggested there was no overall delay in development but strikingly, none of the embryos from the MMI-treated hens hatched, even when incubated up to 24 days (own unpublished results). This latter observation corresponds to what was found for embryos from broiler breeder hens treated with 0.01% PTU in drinking water (36). Also in Japanese quail hens treated with AP, none of the embryos from the high treatment dose (0.4% AP) hatched completely, while embryos from the low dose (0.2% AP) hatched 1 or 2 days late (42). However, we cannot attribute these effects exclusively to a lower maternal TH deposit. Goitrogens are transferred into the egg and taken up by the embryo as shown for instance for MMI (8). One should therefore keep in mind that goitrogens of maternal origin may at least partially inhibit embryonic thyroid gland functioning at later stages. In addition, they can have some direct adverse effects on development, even in early embryos, due to non-TH-related cytotoxicity.

Plasma T_4_ and T_3_ concentrations at E18 and at internal pipping were found to be lower in embryos from PTU-treated broiler breeder hens (36). E14 embryos from AP-treated Japanese quail hens were reported to have decreased body weight as well as decreased thyroidal T_4_ and T_3_ content. Expression of *DIO2* in these embryos was increased in liver but not in brain while expression of another TH-responsive gene, *RC3*/neurogranin, in brain was also unaffected (42). As the data in both studies are from rather late stages of embryonic development, both effects on maternal TH deposit and on embryonic thyroid gland activity may contribute to the observed changes. Sampling of brain and peripheral tissues from embryos of MMI-treated broiler breeder hens at E6, E14, and E18 allowed to show that severe maternal hypothyroidism lowered T_4_ and/or T_3_ levels both prior to and after the start of embryonic thyroid gland functioning. The decrease was in general more pronounced for T_3_ than T_4_, especially in brain (8). This led us to perform also a prospective microarray analysis on extracts of E4 and E8 brain (telencephalon) of embryos from MMI-treated hens. Again, a larger study with more samples is needed to allow detailed analysis, but we identified many differentially expressed genes, both at E4 and E8. Interestingly, only part of the affected genes were identical at the two stages, hinting at the stage-specific effect of THs on brain development (31).

As adequate lowering of TH availability in the early embryo via maternal hypothyroidism is quite labor intensive, including the maintenance of large stocks of goitrogen-treated laying hens, there is a need for alternative methods applicable directly on normal fertilized eggs. One possibility would be to block TH signaling by exposing the embryo to a specific THR antagonist like NH-3 (44). Such an approach has for instance been used successfully to show the severe impact of blocking maternal TH action on neural crest cell migration in early *Xenopus* embryos (45). However, NH-3 can also have some agonistic activities at higher concentrations (46) so it is important to find the right dose for *in vivo* treatment of the species studied. Moreover, in contrast to amphibian eggs, which can take up NH-3 continuously from the surrounding water/medium, the barrier of the avian egg shell implies the need for (probably repeated) injection of the compound. Nevertheless, it would be interesting to test the usefulness of NH-3 or other THR modulators in avian eggs in more detail.

## Effects of Local Knockdown of MCT8 on Neurodevelopment

In recent years our research group opted for an alternative approach, focusing on the TH transporter MCT8. As THs are known to be extremely important for neurodevelopment in all vertebrates and many important steps in chicken central nervous system (CNS) development occur prior to the start of embryonic thyroid gland activity, we chose this target to locally silence *MCT8* gene expression via RNA interference (RNAi) technology. Knockdown of this highly efficient TH transporter that is widely expressed in early chicken CNS (28, 47) allows to reduce cellular uptake of maternal THs and study the consequences for processes such as precursor cell proliferation, migration and differentiation. The MCT8-RNAi vector was generated by cloning synthetic miRNA hairpins within the miRNA operon expression cassette of an pRFPRNAiA vector designed for use in chicken (48, 49) and was transfected into embryonic CNS by electroporation. Changing the timing and site of injection allowed to target specific precursor cell populations that could subsequently be identified by expression of red fluorescent protein (RFP). The fact that knockdown of MCT8 indeed reduced TH signaling in transfected cells (50) and that some of the observed defects could be rescued by supplementation with 3,5,3′-triiodothyroacetic acid (TRIAC), a non-MCT8-dependent TH analog (49), convincingly demonstrated that maternal THs are playing a major role in early CNS development.

**Figure 1:**
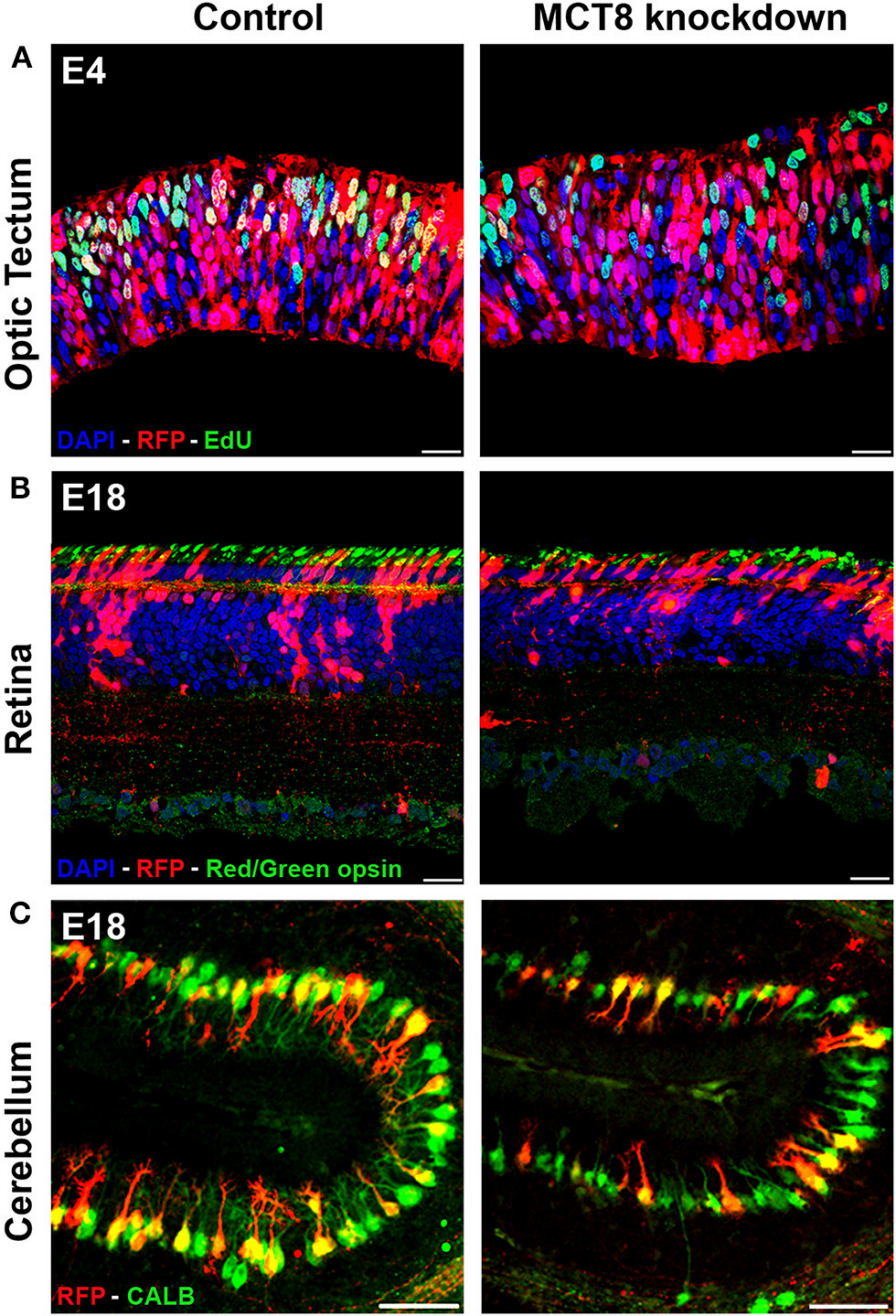
Impact of TH deficiency observed at early and later stages of embryonic chicken CNS development. **(A)** Electroporation of empty vector (control) or MCT8-RNAi vector in the optic tectum at E3 followed by EdU pulse-labeling 1 h before sampling at E4. The strong reduction in the number of proliferating (S phase) transfected cells (yellow) in the knockdown condition illustrates one of the early effects of TH deficiency on CNS development. **(B)** Electroporation of empty vector (control) or MCT8-RNAi vector in the retina at E4 followed by IHC staining for red/green opsin at E18. The lower amount of red/green expressing cones in the mature retina in the knockdown condition at E18 is the combined result of a reduced retinal progenitor cell proliferation and a shift in commitment toward short wavelength sensitive cones at the expense of long/medium wavelength sensitive cones occurring at earlier stages. The picture also shows a reduced thickness of the retina and a disorganization of the sublaminae in the inner plexiform layer in the knockdown condition. **(C)** Electroporation of empty vector (control) or MCT8-RNAi vector in the cerebellar anlage at E3 followed by IHC staining for calbindin (CALB) at E18. The clear reduction in dendritic tree complexity of the Purkinje cells in the knockdown condition may be due to diminished expression of LHX1, LHX5, and RORα, observed at earlier stages. Scale bars represent 20 μm for optic tectum and retina and 100 μm for cerebellum.

Electroporation of the MCT8-RNAi vector into the cerebellar anlage at E3 allowed to knock down MCT8 predominantly in Purkinje cell (PC) precursors. This resulted in a strong decrease in the proportion of LIM homeobox domain transcription factor 1/5 (LHX1/5)-positive cells in the MCT8-RNAi-transfected cell population at E6 compared to similarly treated controls transfected with empty vector (49). This decrease in LHX1/5 protein was accompanied by a decrease in expression of the TH-responsive nuclear receptor retinoic acid receptor-related orphan receptor alpha (*ROR*α). As LHX1, LHX5, and RORα are all very important for early PC differentiation and dendritogenesis (51, 52), these observations were in line with the impaired further development of MCT8-RNAi-transfected PCs, showing a significantly smaller and less complex dendritic tree at E18 (49) (Figure 1C). Importantly, MCT8 deficiency in PCs also induced non-autonomous effects, since it led to reduced granule cell precursor proliferation as shown by reduced incorporation of the proliferation marker 5-ethynyl-2′-deoxyuridine (EdU) in the external germinal layer at E10, and reduced/delayed migration of differentiating granule cells from the external germinal layer to the internal granular layer observed at E18 (49).

Electroporation of the MCT8-RNAi vector in the developing optic tectum at E3 severely disrupted the organization of this layered brain structure. This started with reduced cell proliferation and a premature shift to asymmetric cell divisions in neural progenitors observed at E4, hampering sufficient expansion of the progenitor pool due to precocious neurogenesis (50) (Figure 1A). A second problem, shown by EdU birth-dating experiments between E4 and E12, was impaired migration of both early-born and late-born neuroblasts. This might have been due to the reduced expression of the protein reelin encoded by the TH-responsive gene *RELN* as well as the disorganization of radial glial cell fibers observed at E6 (50). The result of MCT8 deficiency in the optic tectum at E12, a stage where the formation of the different layers in the optic tectum is normally completed (53), was a reduction of optic tectum thickness together with a lower total cell number. This could be linked to the very strong reduction in the multiplication of transfected cells in the MCT8-RNAi condition compared to control-transfected embryos in all different layers of this brain structure (50). In this study too, indications were found for non-autonomous effects, showing a reduction not only in MCT8-RNAi-transfected but also in untransfected GABAergic neurons, a cell type known to depend strongly on TH for its development (50, 54).

Lastly, we also studied the retina, another typically layered structure of the CNS. Knockdown of MCT8 by electroporation at E4 significantly reduced retinal precursor cell proliferation as shown at E6. This resulted in cellular hypoplasia and a thinner retina at E18, where mainly photoreceptors and horizontal cells were lost (55). Although differentiation into retinal ganglion cells and amacrine cells was initially delayed, analysis of the E18 retina showed that the partial loss of some cell types was predominantly due to reduced precursor cell proliferation rather than subsequent differentiation (55). A striking effect of MCT8 deficiency observed at E18, a stage where formation and differentiation of photoreceptors is normally completed (56), was the relative increase in short wavelength-sensitive (UV/blue) cones at the expense of medium/long wavelength-sensitive (red/green) cones (Figure 1B), which is in line with results obtained following deficient TH signaling in murine retina (55, 57, 58). As proliferation of immature photoreceptors occurs predominantly around E6-E8 while opsin expression starts around E14 (56), it can be concluded that the reduction in photoreceptors is the result of a local lack of maternal THs while the shift in cone photoreceptor subtype may be the result of reduced local availability of THs of both maternal and embryonic origin.

Taking advantage of the fact that transfected cells expressed RFP, we also performed fluorescence activated cell sorting (FACS) on cell suspensions of pools of E6 retinas transfected with either MCT8-RNAi or empty vector at E4. RNA isolation of the RFP-positive cell fractions was followed by quantification of expression of a small selection of genes by qRT-PCR. Expression of *THRA* and *THRB2* were respectively 4- and 3-fold lower in MCT8-RNAi transfected cells compared to controls, in line with what is expected in TH-deficient cells. In contrast, expression of *OTX2*, encoding a transcription factor stimulating retinal precursor cells to commit to the photoreceptor cell lineage (59), was 4-fold increased (Vancamp, Houbrechts and Darras, unpublished results). This argues against the possibility that the decreased amount of photoreceptors observed at E18 (see above) was due to a decreased commitment of precursor cells to photoreceptors. Unfortunately, the limited amount of material available did not allow a more in depth analysis, but this approach is certainly worthwhile pursuing in the future.

## Consequences of Variation in Maternal TH Availability on Posthatch Life

Based on the results from studies on chicken embryos prior to the start of embryonic TH production, we now know for sure that a clear reduction in (local) maternal TH availability has strong detrimental effects on early development, while too high levels also have a negative impact (5, 16). Importantly, the studies with MCT8 knockdown also allowed to identify some of the mechanisms involved. As cell proliferation, migration, and differentiation are restricted in time, differing from one tissue/cell population to another, it is clear that many of these defects cannot be corrected at later stages of development when the lack of maternal TH supply may (or may not) be compensated by increased embryonic TH contribution. For example, PCs are the sole output neurons of the cerebellum and are involved in coordinating movement, posture and balance in real-time, but also in long-term motor learning (60). A disrupted cerebellar circuit due to restricted PC arborization and reduced/impaired synaptogenesis with other cerebellar cell types may therefore cause cerebellar ataxia, balance problems and disturbed locomotion (61). Similarly, the defects observed in the chicken retina and in the optic tectum, where visual input is received, processed, and projected to higher brain areas, are likely to have implications for posthatch visual function, including for instance luminance detection and color perception (62). Birds are known to have excellent visual abilities (63) and the connection between the retina and the oculomotor cerebellum via the optic tectum is vital for the control of avian flight (64). The missing link to prove whether the retinal defects observed at E18 have an impact on later life are behavioral studies on posthatch chicks following embryonic MCT8 knockdown. Unfortunately, electroporation in early embryos is a rather invasive technique and so far no hatchlings (control or knockdown) were obtained. Moreover, knockdown by this technique is typically restricted in size, reaching only part of the targeted CNS structure. Knockdown of MCT8 via injection of a viral vector, which is less invasive and allows more widespread knockdown, or the use of CRISPR-Cas9 technology to generate (preferentially conditional) knockout chickens, would provide an important step forward. As mentioned before, injection of a specific THR antagonist would also be an interesting approach, even if repeated injections are be needed to ensure efficient blocking of TH action.

For precocial birds, a literature search only revealed three related papers on the consequences of increased maternal TH availability on posthatch performance of the offspring. They reported that maternal hyperthyroidism via T_4_ supplementation in broiler breeder hens induced some changes in intestinal morphology of male chicks but it did not affect the feed:gain ratio nor the carcass weight at slaughter age (6 weeks) (37). The same chicks seemed to have an increased early adaptive immune response (65) and showed a lower incidence of cold-induced ascites, accompanied by lower hematocrit values compared to cold-exposed controls (36). In all three papers the authors stated that the causal mechanisms remained to be elucidated. The study on the effect of maternal hyperthyroidism in Japanese quail mentioned earlier was not continued until posthatch stages (12) and the same was true for the studies on early TH injection in chicken eggs performed in our own laboratory [(19) and Van Herck and Darras, unpublished results].

There are however two recent reports on the posthatch consequences of TH injection into the eggs of great tits and rock pigeons, altricial species in which the thyroid gland is still immature at hatching. In both studies, a combination of T_4_+T_3_ at slightly elevated physiological doses was injected at the start of incubation. Body weight was decreased in both male and female nestlings in rock pigeon but without concomitant decrease in tarsus length (40). In great tit, both body weight and wing length were decreased in female chicks but increased in male chicks (41). Finally, neither resting metabolic rate nor motor coordination behavior of great tit nestlings seemed to be affected by the treatment nor was the length of the nestling period (41). Since factors such as body weight at fledging are associated with later survival in many birds, both studies indicate that changing maternal TH availability may have an impact on offspring fitness (14). It would have been interesting to have also data on the embryos prior to hatching to find out if any causal links could be found with the changes observed posthatch.

## Conclusion and Future Perspectives

**Figure 2:**
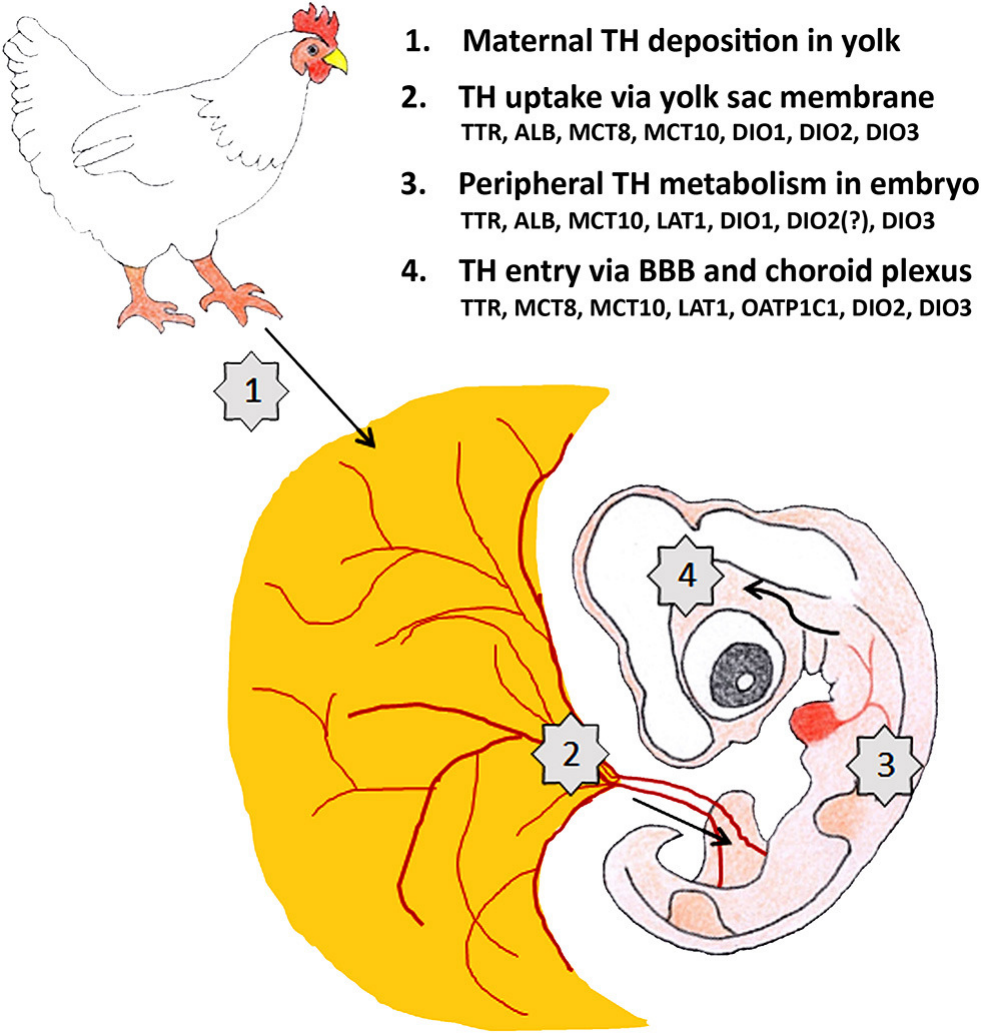
Maternal TH supply to the developing brain of a 4-day-old chicken embryo is regulated at 4 different levels. The factors controlling TH transport and metabolism (TH distributor proteins, TH transporters, deiodinases) shown to be present at E4 at the different levels are mentioned. ALB: albumin, BBB: blood-brain-barrier, DIO1-3: deiodinase 1-3, LAT1: L-type amino acid transporter 1, MCT8-10: monocarboxylate transporter 8-10, OATP1C1: organic anion transporting protein 1C1, TTR: transthyretin.

The stock of maternal THs in avian egg yolk is substantial and is used by the embryo until hatching even if the embryonic thyroid gland becomes active. Maternal THs are therefore important throughout embryonic development not only in altricial but also in precocial birds. Multiple control levels collaborate to fine-tune the amount of T_3_ that finally reaches the THRs in a given tissue (Figure 2). Multiple studies have shown that changes in maternal TH supply have an impact on avian embryonic development while some recent studies point to long lasting effects on posthatch performance and fitness. The challenge for the future is to better understand the link between both observations. From a physiological point of view the focus is on understanding the molecular mechanisms responsible for the observed changes in embryonic development and to link them to changes in posthatch behavior. This can be further investigated in standard laboratory conditions in precocial model species such as chicken and quail, although it is worthwhile to include also comparison with an altricial model species such as laboratory-raised zebra finch. From an ecological point of view it is important to investigate in more detail to what extent environmental factors such as temperature, food, stress, and unfortunately also endocrine disruptors, influence maternal TH deposit in the egg and to find out how this is linked with posthatch fitness of the offspring in changing environmental conditions in both altricial and precocial species.

We should also keep in mind that THs are not the only maternal hormones deposited in avian egg yolk. Other hormones such as sex steroids and corticosteroids are also present and are known to influence embryonic development (66). Moreover, apart from having effects of their own, THs, sex steroids and corticosteroids interact with each other to control development in synergistic as well as antagonistic ways (67, 68). Finally, it is also important to place the results obtained in birds in a broader comparative context. Although it has been debated for quite some time, it is now accepted that THs are needed for early stages of development in all vertebrates. Maternal THs are the only source available for the early embryo, both in mammals and non-mammalian vertebrates, and insights obtained from studies in birds are therefore widely applicable, both in a biological and medical context.

## Author Contributions

The author confirms being the sole contributor of this work and has approved it for publication.

### Conflict of Interest Statement

The author declares that the research was conducted in the absence of any commercial or financial relationships that could be construed as a potential conflict of interest.
